# Antimutagenic Activity and Radical Scavenging Activity of Water Infusions and Phenolics from *Ligustrum* Plants Leaves

**DOI:** 10.3390/molecules14010509

**Published:** 2009-01-22

**Authors:** Milan Nagy, Lívia Križková, Pavel Mučaji, Zuzana Kontšeková, František Šeršeň, Juraj Krajčovič

**Affiliations:** 1 Department of Pharmacognosy and Botany, Faculty of Pharmacy, Comenius University, Odbojarov 10, 83232 Bratislava, Slovak Republic; E-mails: mucaji@fpharm.uniba.sk (P. M.), kontsekova@fpharm.uniba.sk (Z. K.); 2 Institute of Cell Biology, Faculty of Natural Sciences, Comenius University, Mlynská dolina, 84215 Bratislava, Slovak Republic; E-mails: krizkoval@ba.netlab.sk (L. K.), krajcovic@fns.uniba.sk (J. K.); 3 Institute of Chemistry, Faculty of Natural Sciences, Comenius University, Mlynská dolina, 84215 Bratislava, Slovak Republic; E-mail: sersen@ fns.uniba.sk (F. Š.).

**Keywords:** *Ligustrum*, *Euglena gracilis*, Phenolics, Antimutagenicity, Lipophilicity, DPPH

## Abstract

Water infusions of *Ligustrum delavayanum* and *Ligustrum vulgare* leaves and eight phenolics isolated therefrom have been assayed *in vitro* on ofloxacin-induced genotoxicity in the unicellular flagellate *Euglena gracilis*. The tested compounds luteolin, quercetin, luteolin-7-glucoside, luteolin-7-rutinoside, quercetin-3-rutinoside, apigenin-7-rutinoside, tyrosol and esculetin inhibited the mutagenic activity of ofloxacin (43 µM) in *E. gracilis.* Water infusions from leaves of *L. delavayanum* and *L. vulgare* showed higher antimutagenic effect (p_t_ < 0.001). The activity of these samples against ofloxacin (86 µM)-induced genotoxicity was lower, but statistically significant (p_t_ < 0.05), excluding the water infusion of *L. delavayanum* leaves (p_t_ < 0.01). Efficacy of quercetin, luteolin-7-rutinoside, apigenin-7-rutinoside was insignificant. The antimutagenic effect of most phenolics we studied could be clearly ascribed to their DPPH scavenging activity, substitution patterns and lipophilicity.

## Introduction

There is a continuing interest in naturally occurring compounds widely distributed in the plant kingdom, particularly in those having potential utility due to their therapeutical properties. Some species from the genus *Ligustrum spp.* have been used in traditional Chinese and Japanese medicine for centuries, mostly because of their anticancerogenic, immunomodulatory [1], cardioprotective [2], antibacterial [3] or antidiabetic effects [4]. It is suggested that their biological effects are caused by the presence of glycosides namely, flavonoids, phenylpropanoids, terpenoids (mainly secoiridoids), and their aglycones [5,6,7].

The aim of this study was to assess the potential antimutagenic activity of eight phenolic compounds isolated from *Ligustrum* plants against the genotoxicity of mutagenic agent ofloxacin using the *Euglena gracilis* assay. The photosynthetic unicellular flagellate *E. gracilis* possesses a multigenomic system with nuclear, mitochondrial and chloroplast DNAs. The chloroplast genome is particularly sensitive to various chemical and physical factors resulting in the degradation or complete loss of chloroplast DNA [8,9]. The hereditary loss of functional chloroplasts is evidenced by the formation of heterotrophic colourless colonies. As we reported in our reproducible findings described in series of studies, the *E. gracilis* is a suitable eukaryotic model for investigation of mutagenesis and antimutagenesis [8,9,10,11,12].

The antiradical properties of phenolic compounds were measured using the 1,1-diphenyl-2-picrylhydrazyl (DPPH) assay – the most often used method for determination of radical scavenging activity. In many studies carried out on isolated plant phenolics [13,14] or complex natural mixtures [15,16] the method was used to describe the kinetic behaviour or different reaction mechanisms of antioxidant activity. Correlation between DPPH radical scavenging and antimutagenic activity of plant phenolics in *Salmonella* assay was also studied [17,18]. The present work describes, to our best knowledge, the first study on the antimutagenic and DPPH radical scavenging activity of water infusions and phenolics isolated from *Ligustrum delavayanum* (LD) and *L. vulgare* (LV) using an *E. gracilis* assay.

## Results and Discussion

The antimutagenic activity of the phenolics was evaluated in experiments involving application of genotoxic agent ofloxacin to *E. gracilis*, which resulted in the formation of mutant (white) colonies. In control plates, no incidence of white colonies was observed. Likewise, application of phenolic compounds did not cause formation of mutant colonies of *E. gracilis*. No change in cell viability was observed. The presence of DMSO in separate control incubations with mutagen ofloxacin did not influence the mutagenic activity of ofloxacin (data not shown). On the other hand, application of ofloxacin led to marked incidence of mutations. Ofloxacin at a concentration of 43 µM induced 80.1 ± 6.3% of irreversible white mutant cells, and at the concentration of 86 µM, it induced 99.6 ± 1.0% of mutants. The viability of the *Euglena* cells was estimated by counting the total colony cell number affected by mutagen compared to that of the control plate. The viability of *Euglena* cells after ofloxacin treatment did not change when compared to the negative control.

**Figure 1:**
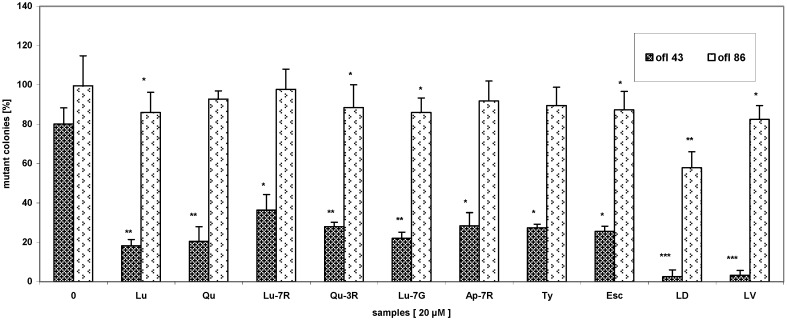
Effect of phenolic compounds and water infusions on ofloxacin-induced mutagenicity in *E. gracilis*. Each value is average ± SD of four separate experiments, nine plates together, *p_t_ < 0.05, **p_t_ < 0.01, ***p_t_ < 0.001 (statistical significance for individual samples compared to those of the control), p_A_ < 0.001, ofloxacin 43 µM and 86 µM, compounds (20 μM), water infusions (equivalent to 20 μM pure compounds samples concentrations).

The 43 µM ofloxacin-induced bleaching was effectively inhibited by the presence of phenolics and infusions. The highest protective effect on the mutagenicity of ofloxacin showed the water infusions of LD and LV and the proportion of white colonies decreased from 80.1 ± 6.3% to 2.5 ± 3.5% and 3.2 ± 2.5%, respectively, which represents about 96.8% and 96.0% reduction of the initial mutant incidence in water infusions untreated plates. The rest of phenolics have also showed notable statistically significant effects compared to those of the control (*t*-test p_t_ < 0.05 – 0.001, ANOVA test p_A_ < 0.001) (Figure 1).

In the case of the 86 µM ofloxacin-induced mutagenicity, the water infusion of LD again showed the highest antimutagenic potency, followed by the infusion of LV and the number of white colonies decreased from 99.6 ± 1.0% to 57.9 ± 7.3% and 82.5 ± 7.0%, which implies a relative decrease of about 41.8% and 17.2% (pt < 0.01 - 0.05), respectively. The activity of both luteolin and luteolin-7-glucoside caused reduction of the number of white colonies from 99.6 ± 1.0% to 86.0 ± 4.3%, and of tyrosol to 89.5 ± 6.4% (pt < 0.05). The antimutagenic potency of apigenin-7-rutinoside, quercetin and luteolin-7-rutinoside was less than 10% (p_t_ > 0.05) (Figure 1).

Cumulative data of the radical scavenging activity (SC_50_), the antimutagenic potency of tested samples against both ofloxacin concentrations, 43 µM and 86 µM, and LogKow values are given in the Table 1.

**Table 1:** Antimutagenic potency of phenolics and water infusions from *Ligustrum* plants leaves against ofloxacin-induced mutagenicity in *E. gracilis,* radical scavenging activity (SC_50_) value (μM) of phenolic compounds (20 μM) and water infusions (equivalent to 20 μM pure compounds samples concentrations), and lipophilicity (LogKow values), n/a = not available data.

Sample	Ofloxacin43 µM	Ofloxacin86 µM	Scavenging activity	Lipophilicity
	Antimutagenic potency (%)		SC_50_ (μM)	LogKow
Lu	77.3	13.6	6.7	2.36
Qu	74.4	6.8	9.2	1.82
Lu-7R	54.7	1.8	13.4	-0.79
Qu-3R	65.2	11.2	14.2	-0.25
Lu-7G	72.3	13.6	12.6	0.35
Ap-7R	64.5	7.7	425.2	-0.31
Ty	65.7	10.2	6, 900.0	1.09
Esc	68.2	12.3	8.6	0.55
LD	96.8	41.8	97.3	n/a
LV	96.0	17.2	48.1	n/a

The highest scavenging activity against DPPH radical was exhibited by luteolin, esculetin and quercetin, relatively non-polar substances with *ortho*-dihydroxy phenolic groups. A decrease of the SC_50_ value (= scavenging activity increase) depends on an increase of the phenolic’s lipophilicity due to attached saccharide units, cf. LogK_ow_ values for luteolin > luteolin-7-glucoside > luteolin-7-rutinoside, or quercetin > quercetin-3-rutinoside. Isolates without *ortho*-dihydroxy phenolic groups, i.e. apigenin-7-rutinoside, a relatively polar compound, and tyrosol, a relatively non-polar compound, exhibited the lowest scavenging activity. The *ortho*-dihydroxy substitution in the B-ring seems to be the main requisite for the scavenging activity because of oxidation of the flavonoid takes place in ring B for catechol-containing derivatives [19,20,21]. Furthermore, scavenging activities of both LV and LD water infusions were about 10-fold lower than the activity of the most active isolates (luteolin, esculetin, quercetin), and about 10- and 17-fold higher than the two substances with the lowest scavenging activity (apigenin-7-rutinoside and tyrosol). Probably other unknown active minor constituent(s) and/or another mechanism(s) could be responsible for the high antimutagenic potency of these infusions.

At a 43 μM ofloxacin concentration the antimutagenic activity correlates with LogK_ow_ values for samples with *ortho*-dihydroxy substitution patterns, e.g. aglycone homologues (luteolin > luteolin-7-glucoside > luteolin-7-rutinoside or quercetin > quercetin-3-rutinoside). This also ocurrs for all tested flavonoids with an *ortho*-dihydroxy substitution in the B-ring (luteolin > quercetin > luteolin-7-glucoside > quercetin-3-rutinoside> luteolin-7-rutinoside). The same situation was observed earlier for flavonoids [22,23]. At an 86 μM ofloxacin concentration however, increased antimutagenicity of the Q-3R sample does not correlate with its lipophilicity ranking.

Antimutagenic effect of the phenolics was observed in experiments performed with ofloxacin, a DNA gyrase-inhibitor [24] and a producer of reactive oxygen species (ROS) [10,11,25]. The obtained results showed that phenolic compounds and infusions exhibited noticeable, statistically significant, antimutagenic activity on ofloxacin (43 µM)-induced damage to the chloroplast DNA of *E. gracilis.* In the case of a higher (86 µM) ofloxacin concentration-induced damage of the *E. gracilis* chloroplast DNA the antimutagenic activity was lower or statistically insignificant. These results are very similar to the ones describing the effect of flavonoids as previously published [11]. We had demonstrated that fluoroquinolones produce superoxide anion, which can cause an electron transfer imbalance in chloroplasts resulting in their damage through oxidative stress. Therefore we hypothesize, that the antimutagenic potency of tested compounds may be based on their ability to prevent the oxidative damage of DNA by ROS. The antioxidants interact with various radical species and decrease the induction of DNA-damage and subsequent mutations by scavenging ROS [26].

At an ofloxacin concentration of 43 μM the antimutagenic activity correlates positively with the DPPH scavenging activity for samples with *ortho*-dihydroxy substitution patterns – once again with an exception of the Q-3R sample. The reasons for this exception need to be more carefully studied.

Both, the LV and LD infusions were relatively weak scavengers, but very strong antimutagens at the 43 µM ofloxacin concentration, while at the 86 µM ofloxacin concentration only the LD infusion showed notable antimutagenic potency (41.8 %, p_t_ < 0.01). One can assume that a two-fold higher ofloxacin concentration (86 *vs*. 43 µM) causes approximately the same increase of free radicals in tested samples. The above mentioned high activity of the LD sample could be explained by the different content of active agents and/or their ratios in LD and LV infusions, as confirmed by HPLC analyses (unpublished results on file – paper in preparation). An explanation may be that a sufficient concentration of active substances is present only in the LD sample. Thus, more free radicals can be effectively scavenged and a following substantial antimutagenic effect can be observed. Analogically could be a weak antimutagenicity – scavenging activity correlation of some phenolics at 86 µM explained.

An Ames test with *Salmonella typhimurium* TA102 established that the correlation between the antimutagenic properties of flavonoids and their DPPH radical scavenging activity was structure dependent [17]. Flavones or flavonols with a ring B *ortho*-dihydroxy structure were the best scavengers and antimutagens. Glycosidation of the hydroxyl group at C-3, C-7 or monohydroxy substitution in ring B decreased the antimutagenic activity, which was not linearly related to the decreased scavenging activity. This statement correlates with our results. Another study determining the relationship of chemical structure and antimutagenic activity of various tea extracts in the *Salmonella typhimurium* TA100 had shown that the inhibitory effect against the mutagenicity of 2-amino-3-methylimidazo (4,5-f)quinoline (IQ) and 2-amino-6-methyldipyrido(1,2-a:3',2'-d)imidazole (Glu-P-1), was related to their content of catechins and ascorbic acid. However, no significant correlation was found between the antimutagenic activity of the tea extracts to benzo[α]pyrene (B[α]P) or aflatoxin B1 (AFB1) and the content of major tea extracts components [27]. The antioxidant effect of various tea extracts correlated, in some cases well, with their antimutagenic activity, but varied with the mutagen and the antioxidative properties of tea extracts. Correlation coefficients between the scavenging effect of DPPH radical and the antimutagenicity of tea extracts in *S. typhimurium* TA98 were not same for all scavengers [28]. Namely, in some cases the correlation coefficients were 0.90 against IQ, 0.86 and 0.82 against AFB1 and Glu-P-1, but only 0.54 and 0.50 against B[α]P and Trp-P-1 (3-amino-1,4-dimethyl-5*H*-pyrido[4,3-*b*]indole) mutagens. Contrary to that the correlation coefficients between the scavenging effect of DPPH radical and antimutagenic activity of tea extracts to all used mutagens, performed with *S. typhimurium* TA100, were only 0.47 against IQ; 0.45 against (B[α]P); 0.69 against AFB1and Glu-P-1, and 0.04 against Trp-P-1. These results showed that Ames´ tests did not produce homogenous results. So, the assay we used is a suitable alternative to the Ames assay because of the multigenomic system with nuclear, mitochondrial and chloroplast DNAs of *E. gracilis*. In a different test system, performed with *E. coli,* out of the five plant samples that positively reduced the DPPH radical and inhibited lipid peroxidation, only one of them was shown to be an effective antimutagen [29]. Both, the antimutagenic and anticarcinogenic activity of polyphenols is mostly due to their antioxidant activity, which inactivates direct mutagens/carcinogens and inhibits the activation of indirect mutagens/carcinogens extracellularly. Polyphenols also enhance the level of cellular antioxidative system and induce the cytochrome P-450 resulting in detoxifying the activity of carcinogens intracellularly [30].

In summary, the antimutagenic effect of flavonoids we have now studied can be ascribed particularly to their *ortho*-dihydroxy substitution patterns on ring B and lipophilicity, and to a lesser extent, to scavenging properties. However, determined suppression of mutagenic effect can be achieved also due to other simultaneous mechanism(s) operating in reducing of mutagenic effect of ofloxacin, e.g. metal ion complexation, reducing power capacity, ROS and/or RNS scavenging.

## Experimental

### Chemicals

Ofloxacin (purity > 99.5%) was purchased from Sigma-Aldrich Chemie GmbH (Steinheim, Germany). DMSO was obtained from Merck (Darmstadt, Germany), methanol and DPPH radical from Sigma (Salt Lake City, USA). Fresh solution of ofloxacin was prepared by dissolving it in 0.1 M NaOH. Stock solutions of phenolics were prepared in DMSO and kept at 10 °C in the dark.

### Plant material

Leaves from *Ligustrum delavayanum* Hariot and *Ligustrum vulgare* L. (Oleaceae) collected in the Arboretum Mlyňany, Dendrobiology Institute of Slovak Academy of Sciences in May 2005, dried at room temperature, and stored at 4 °C. Voucher specimens are deposited at the corresponding author’s laboratory. The phenolics luteolin (Lu), quercetin (Qu), luteolin-7-rutinoside (= rhamnoglucosyl, Lu-7R), quercetin-3-rutinoside (= rhamnoglucosyl, Ru), apigenin-7-rutinoside (= rhamnoglucosyl, Ap-7R), luteolin-7-glucoside (Lu-7G), *p-*hydroxyphenethylalcohol (= tyrosol, Ty) and esculetin (Esc) were previously isolated from the aforementioned plant sources and their structures were determined by spectroscopic means [31,32]. Water infusions made from leaves of LD and LV were prepared by hot distilled water maceration (15 min, 1:10 plant-water weight ratio), and filtration after cooling. Total concentration of flavonoids in infusions was expressed as quercetin equivalents (data not shown) and set equivalent to 20 µM of pure compounds samples concentrations in the mutagenicity assay. Pure phenolics were used at the concentration of 20 µM in all experiments as well as in controls. The concentration of DMSO in cultivation medium never exceeded 0.4%.

### Euglena gracilis assay

*E. gracilis* strain Z (1224-5/90) was obtained from Sammlung von Algenkulturen (Göttingen, Germany). The cells were cultivated and the bleached mutants were evaluated as described earlier [11]. *Euglena* cells were co-treated with 43 μM or 86 μM of ofloxacin plus 20 μM of each phenolic compound or water infusion at 27 °C and a constant light of 2000 lux. Following a 24 hours co-treatment the cells were washed out, diluted and spread on agar plates with Cramer-Myers medium (1.2% agar). Green (normal) and white (mutant) colonies were analyzed after 10 - 14 days of cultivation in the light at 27 °C. Solutions of each phenolic compound at 20 μM concentration were used as control samples without addition of ofloxacin. Three plates were used in each cultivation experiment for each phenolic compound and water infusions. The experiments were repeated in four independent series and statistically analyzed. The numbers of bleached and green colonies were counted and their percentages were estimated. The relative decrease of the ofloxacin- induced bleaching as the antimutagenic potency (AP) of the applied phenolics was calculated according to the eq. (1):

(%) AP = [(B_o_ –B_p_)/B_o_] x 100 (1)

where AP is the antimutagenic potency (%), B_o_ is the ofloxacin- induced *Euglena* bleaching (%), and B_p_ is the ofloxacin- induced and phenolics- reduced *Euglena* bleaching (%).

### DPPH assay

The DPPH assay was performed according to [33]. Briefly, samples (200 µL, phenolics concentrations 1–100 µM, water infusions 1–200 μM, equivalent to phenolics samples concentrations) and methanolic DPPH solution (1800 µL, 55 µM) were combined and kept in the dark at 37 °C for 30 minutes. The absorbance of samples was measured at 517 nm on a Spectronic Genesys 6 apparatus. A concentration dependent decrease of the DPPH initial (= 100 %) absorbance value was used for SC_50_ (µM) (= the concentration needed for 50% absorbance decrease) calculation using CompuSyn 1.0.1 software (ComboSyn Inc., 1277 Paramus, NJ, USA). The DPPH control (containing no sample) was prepared using the same procedure. Six measurements for each sample were performed.

### Statistical analysis

The statistical significances of all calculated values were determined by paired Student´s t-test (p_t_) and variance analysis ANOVA (F-test) (p_A_). The values represent the means ± standard deviation (SD).

### LogK_ow_ calculation

The free service available on *http://www.syrres.com/esc/est_kowdemo.htm* was used for all tested isolates.

## References

[1] Baróniková S., Nagy M., Grančai D. (1999). Changes in immunomodulatory activity of human mononuclear cells after cultivation with leaf decoctions from the genus *Ligustrum* L.. Phytother. Res..

[2] Yim T.K., Wu W.K., Pak W.F., Ko K.M. (2001). Hepatoprotective action in an oleanolic acid-enriched extracts of *Ligustrum lucidum* fruits is mediated through an enhancement on hepatic glutathione regeneration capacity in mice. Phytother. Res..

[3] Jantová S., Nagy M., Ružeková Ľ., Grančai D. (2000). Antibacterial activity of plant extracts from the families Fabaceae, Oleaceae, Philadelphaceae, Rosaceae and Staphyleaceae. Phytother. Res..

[4] Andrade-Cetto A., Henrich M. (2005). Mexican plants with hypoglycaemic effect used in the treatment of diabetes. J. Ethnopharmacol..

[5] Pieroni A., Pachaly P., Huang Y., van Poel B., Vlietinck A.J. (2000). Studies on anti-complementary activity of extracts and isolated flavones from *Ligustrum vulgare* and *Phillyrea latifolia* leaves (Oleaceae). J. Ethnopharmacol..

[6] Ma S.C., He Z.D., Deng X.L., But P.P., Eng-Choon O.V., Xu H.X., Hon-Sun Lee S., Lee S.F. (2001). In vitro evaluation of secoiridoid glucosides from the fruits of *Ligustrum lucidum* as antiviral agents. Chem.Pharm. Bull..

[7] He Z.D., Lau K.M., But P.P., Jiang R.W., Dong H., Ma S.C., Fung K.P., Ye W.C., Sun H.D. (2003). Antioxidative glycosides from the leaves of *Ligustrum robustum*. J. Nat. Prod..

[8] Križková L., Horniak L., Sláviková S., Ebringer L. (1996). Protective effects of sodium selenite on ofloxacin-induced loss of chloroplast DNA in *Euglena gracilis*. Folia Microbiol..

[9] Krajčovič J., Ebringer L., Schwartzbach S.D., Seckbach J. (2002). Symbiosis: Mechanisms and Model Systems.

[10] Ebringer L., Dobias J., Krajčovič J., Polónyi J., Križková L., Lahitová N. (1996). Antimutagens reduce ofloxacin-induced bleaching in *Euglena gracilis*. Mutat. Res..

[11] Križková L., Nagy M., Polónyi J., Ebringer L. (1998). The effect of flavonoids on ofloxacin-induced mutagenicity in *Euglena gracilis*. Mutat. Res..

[12] Križková L., Nagy M., Polónyi J., Dobias J., Belicová A., Grančai D., Krajčovič J. (2000). Phenolic acids inhibit chloroplast mutagenesis in *Euglena gracilis*. Mutat. Res..

[13] Bondet V., Brand-Williams W., Berset C. (1997). Kinetics and mechanisms of antioxidant activity using the DPPH free radical method. Lebensm.-Wiss. u.-Technol..

[14] Hotta H., Nagano S., Ueda M., Tsujino Y., Koyama J., Osakai T. (2002). Higher radical scavenging activities of polyphenolic antioxidants can be ascribed to chemical reactions following their oxidation. Biochim. Biophys. Acta.

[15] Cevallos-Casals B.A., Cisneros-Zevallos L. (2003). Stoichiometric and Kinetic Studies of Phenolic Antioxidants from Andean Purple Corn and Red-Fleshed Sweetpotato. J. Agric.Food Chem..

[16] Sendra J.M., Sentandreu E., Navarro J.L. (2006). Reduction kinetics of the free stable radical 2,2-diphenyl-1-picrylhydrazyl (DPPH) for determination of the antiradical activity of citrus juices. Eur.Food Res.Technol..

[17] Edenharder R., Grünhage D. (2003). Free radical scavenging abilities of flavonoids as mechanism of protection against mutagenicity induced by *tert*-butyl hydroperoxide or cumene hydroperoxide in *Salmonella typhimurium* TA102. Mutat. Res..

[18] Wozniak D., Lamer-Zarawska E., Matkowski A. (2004). Antimutagenic and antiradical properties of flavones from the roots of *Scutellaria baicalensis* Georgi. Nahrung.

[19] van Acker S.A.B.E., De Groot M.J., Van Berg D.J.D., Tromp M.N.J.L., Kelder G.D.O.D., Van Der Vijgh W.J.F., Bast A. (1996). A quantum chemical explanation of the antioxidant activity of flavonoids. Chem. Res. Toxicol..

[20] Sekher Pannala A., Chan T.S., O'Brien P.J., Rice-Evans C.A. (2001). Flavonoid B-ring chemistry and antioxidant activity: Fast reaction kinetics. Biochem. Biophys. Res. Commun..

[21] Burda S., Oleszek W. (2001). Antioxidant and antiradical activities of flavonoids. J. Agric. Food Chem..

[22] Plumb G.W., Price K.R., Williamson G. (1999). Antioxidant properties of flavonol glycosides from tea. Redox Rep..

[23] Williamson G., Plumb G.W., Garcia-Conesa M.T. (1999). Glycosylation, esterification and polymerization of flavonoids and hydroxycinnamates: effects on antioxidant properties. Basic Life Sci..

[24] Hooper D.C., Wolfson J.S., Hooper D.C., Wolfson J.S. (1993). Quinolone Antimicrobial Agents.

[25] Umezawa N., Arakane K., Ryu A., Matshiko S., Hirobe N., Nagano T. (1997). Participation of reactive oxygen species in photoxicity induced by quinolone antibacterial agents. Arch. Biochem. Biophys..

[26] Slameňová D., Lábaj J., Križková L., Kogan G., Šandula J., Bresgen N., Eckl N.P. (2003). Protective effects of fungal (1→3)-β-D-glucan derivatives against oxidative DNA lesions in V79 hamster lung cells. Cancer Lett..

[27] Yen G.C., Chen H.Y. (1996). Relationship between antimutagenic activity and major components of various teas. Mutagenesis.

[28] Yen G.C., Chen H.Y. (1995). Antioxidant activity of various tea extracts in relation to their antimutagenicity. J. Agric. Food Chem..

[29] Ramos A., Visozo A., Piloto J., Garcia A., Rodriguez C.A., Rivero R. (2003). Screening of antimutagenicity via antioxidant activity in Cuban medicinal plants. J. Ethnopharmacol..

[30] De Flora S. (1998). Mechanisms of inhibitors of mutagenesis and carcinogenesis. Mutat.Res..

[31] Nagy M., Baróniková S., Grančai D., Mučaji P. (2001). Constituents of *Ligustrum delavayanum* Hariot leaves. (in Slovak). Česká a Slov. Farm..

[32] Šeršeň F., Mučaji P., Grančai D., Nagy M., Švajdlenka E. (2006). Constituents of butanol extract from leaves of *Ligustrum vulgare* L.. Acta Facult. Pharm. Univ. Comenianae.

[33] Molyneux P. (2004). The use of the stable free radical diphenylpicrylhydrazyl (DPPH) for estimating antioxidant activity. J. Sci. Technol..

